# Risk factors for immediate and delayed cardiogenic shock in patients with ventricular septal rupture after myocardial infarction

**DOI:** 10.3389/fcvm.2023.1230169

**Published:** 2023-11-23

**Authors:** Si Wang, Jing Zhang, Qian-Feng Xiao, Kai Liu, Ying Xu, Xiao-Ping Chen, Xin Wei, Yong Peng

**Affiliations:** Department of Cardiology, West China Hospital, Sichuan University, Chengdu, China

**Keywords:** risk factors, immediate, delayed, cardiogenic shock, ventricular septal rupture, myocardial infarction

## Abstract

**Background:**

Ventricular septal rupture (VSR) is a serious complication occurring after myocardial infarction (MI). Cardiogenic shock (CS) is a common complication of VSR and an important factor affecting its prognosis. CS can occur in either an immediate or delayed manner after VSR; however, studies on the risk factors associated with immediate or delayed CS are scarce.

**Methods:**

We retrospectively studied patients diagnosed with VSR after MI and admitted to the West China Hospital between September 2009 and August 2023. Demographic data, medical history, physical examination results, electrocardiograms, and echocardiographic and hematological data were extracted from electronic medical records or archived records. CS was defined as hypotension (<90 mmHg) and/or the requirement for catecholamines, pulmonary congestion, and signs of end-organ failure. The CS onset time was defined as the time at which catecholamines were initiated.

**Results:**

A total of 88 patients with VSR after MI, including 49 males (55.7%), were enrolled. The average age was 70.2 years. Of these patients, 32 (36.4%) who already had CS at the time of VSR discovery were defined as immediate CS, and 28 (31.8%) who developed CS within 2 weeks after VSR discovery were defined as delayed CS. A smaller left ventricular end-diastolic diameter (LVEDD) and VSR discovered after admission were independent risk factors for immediate CS. Elevated heart rate and higher levels of creatine kinase-MB isoenzyme on admission were independent risk factors for delayed CS in patients without immediate CS after VSR.

**Conclusions:**

The occurrence of CS in patients with VSR after MI has an evident time course. Thus, an early identification of patients at risk of immediate or delayed CS and optimization of treatment procedures may help improve the prognosis.

## Introduction

Ventricular septal rupture (VSR) is a serious complication occurring after myocardial infarction (MI), with a reported incidence rate of 0.2%–0.3% in the era of thrombolysis and primary percutaneous coronary intervention (PCI) (1, 2). Repairing the ruptured septum is considered the most vital treatment method since the survival rate following conservative drug treatment is less than 10% (1, 3, 4). Owing to the fragility of the necrotic myocardium in the acute phase of MI, the mortality and re-rupture rates are high after early repair (5). Although the optimal repair time has not been determined, prolonging the preoperative waiting time can improve the success rate of repair in patients with relatively stable hemodynamics (6–8). Cardiogenic shock (CS) is a common complication of VSR, and it is considered to be an important cause of poor prognosis in patients with VSR (7, 9, 10). It can occur in an immediate or delayed manner following VSR and lead to multiple organ dysfunction or even hemodynamic collapse requiring emergency repair (7). Identifying patients with a high risk of developing immediate or delayed CS after VSR and optimizing treatment procedures may help prolong the preoperative time and improve the prognosis of these patients. However, studies on the risk factors of immediate or delayed CS after VSR are scarce. This study aims to elucidate the risk factors for immediate and delayed CS in patients with VSR after MI to provide a reference for clinical practice.

## Materials and methods

### Study population and related definitions

We retrospectively studied patients diagnosed with VSR after MI who were admitted to the West China Hospital between September 2009 and August 2023. All patients with discharge diagnose, including MI and VSR, were included in the study. Patients with ventricular septal defects due to endocarditis, congenital heart disease, or trauma were excluded. Data were extracted from electronic medical or archived records. History of smoking, height, weight, systolic blood pressure (SBP), diastolic blood pressure (DBP), and heart rate were obtained from the admission records. Hypertension, diabetes mellitus, history of coronary heart disease, and pneumonia were derived from the discharge diagnose. CS was defined as hypotension (<90 mmHg) and/or the requirement for catecholamines, pulmonary congestion, and signs of end-organ failure (11, 12). The occurrence of CS in our study was determined by two experienced physicians based on blood pressure (BP), clinical manifestations, lactate levels, and documented administration of catecholamines in the medical records. The onset time of CS was defined as the time of the initiation of administering catecholamine, including epinephrine, norepinephrine, and dopamine. Since delayed VSR repair is usually performed more than 2 weeks after MI as reported in many studies (13), we focus on the risk factors for CS with 2 weeks after VSR. We defined patients who already had CS at the time of VSR discovery as immediate CS, other patients who developed CS within 2 weeks after VSR discovery despite hemodynamic stability at the beginning were defined as delayed CS, and those who did not develop CS with 2 weeks after VSR discovery were identified as non-CS. The location of the MI was determined according to the electrocardiogram and discharge diagnose, which included anterior and inferior MI. Echocardiographic data were obtained when VSR was first detected at our hospital, and hematological data were obtained upon admission. Mitral and tricuspid valve regurgitation were defined as moderate or severe regurgitation. Primary PCI was defined as revascularization performed within 12 h after the onset of MI. Preoperative PCI referred to the revascularization therapy prior to VSR repair. Coronary artery stenosis was defined as a degree of stenosis exceeding 70% of the lumen. Inotrope use indicated the use of amrinone, milrinone, dobutamine, or levosimendan. Mechanical circulatory support (MCS) included intra-aortic balloon pump (IABP) and extracorporeal membrane oxygenation. The ventricular septal repair included surgical and transcatheter repairs. Due to regional customs, some patients were discharged automatically when CS could not be reversed by medications or MCS and died within a short time after discharge. Therefore, survival was defined as a patient surviving ≥30 days after discharge. Survival information after discharge was collected via the telephone. The time of MI was defined as the time when the patient first experienced chest pain for ≥30 min during their visit, which was determined by two experienced physicians referring to the medical records and the values of creatine kinase-MB isoenzyme (CK-MB) and troponin-T on admission. If the VSR was discovered prior to admission, the time of VSR discovery was determined as the time when the VSR was first detected by echocardiography in another hospital, as recorded in the medical records. If the VSR was discovered after admission, the time of VSR discovery was based on the time when the cardiac murmur was first recorded in the medical records or the time when VSR was first detected by echocardiography in our hospital if there was no cardiac murmur recorded in the medical records. Patients who did not have VSR at the time of CS but develop VSR subsequently were excluded. Those patients who were discharged in a stabilized condition but died outside the hospital within 2 weeks after VSR discovery were excluded from the analysis because we could not determine whether these patients would develop CS if they continued to be hospitalized. Those patients who were in a stabilized condition 2 weeks after VSR discovery but died eventually were included in the non-CS group. This study was approved by the ethics committee of the West China Hospital, Sichuan University (Approved No. of ethic committee 2021–1770).

### Statistical analysis

Continuous variables were presented as means and standard deviations or as medians and quartiles, as appropriate. Categorical variables were presented as frequencies and percentages. One-way analysis of variance (ANOVA) or Wilcoxon rank-sum test was used to compare categorical variables among the three groups, and the least significant difference (LSD) test was used for pairwise comparisons between groups in ANOVA analysis. Categorical variables were compared using Pearson's chi-square test or Fisher's exact test. Univariate and multivariate logistic regression analyses (forward: LR) were used to compare odds ratios (ORs) with 95% confidence intervals (CIs) to assess the independent risk factors for immediate CS in patients with VSR after MI compared with patients without immediate CS and for delayed CS in patients without immediate CS after VSR compared with patients in the non-CS group. Age, sex, and variables with a statistical correlation with immediate or delayed CS in the univariate logistic regression analysis were included in the multivariable logistic regression analysis. The SPSS software package (version 26.0; SPSS, Chicago, IL, USA) was used for statistical analysis. Statistical significance was defined as *P* < 0.05.

## Results

### Basic information of the patients

**Figure 1:**
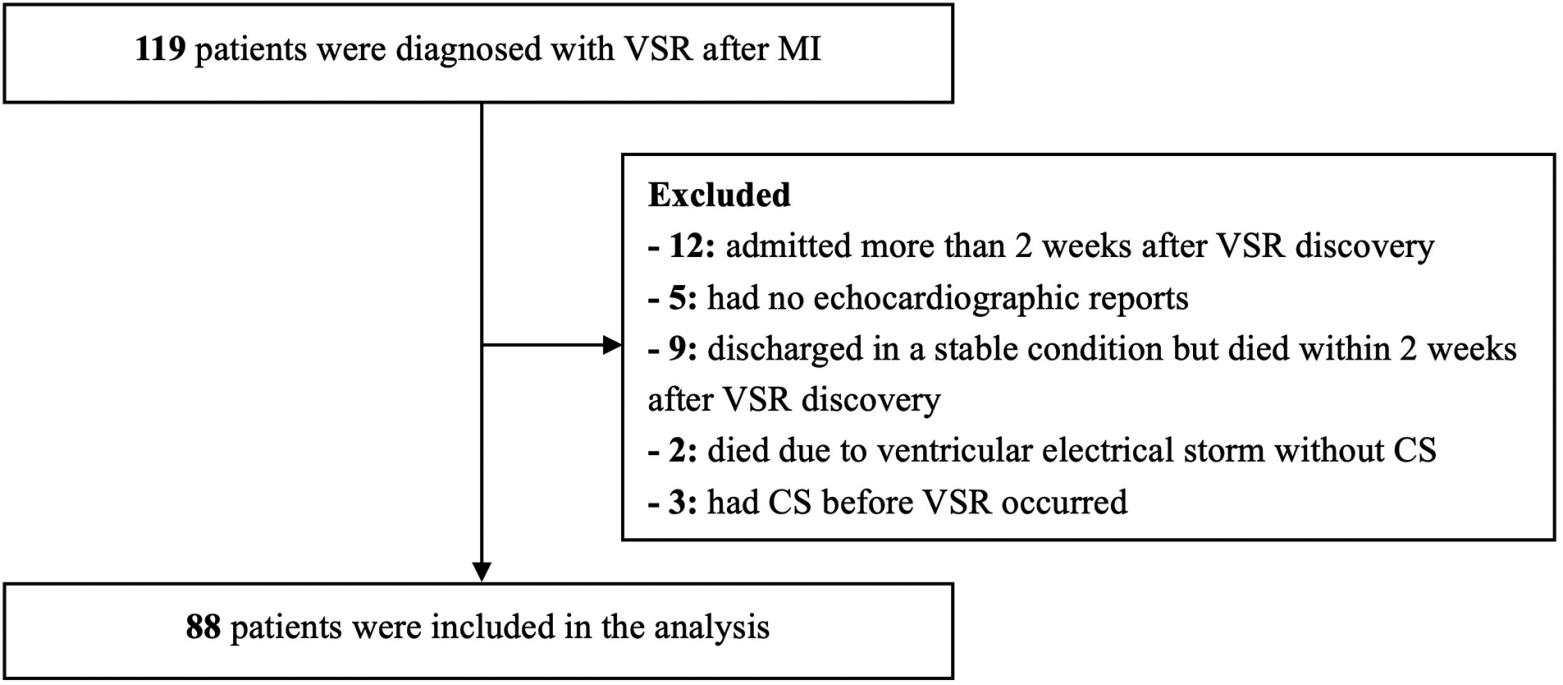
The flow chart of the study. VSR, ventricular septal rupture; MI, myocardial infarction; CS, cardiogenic shock.

Of the 119 patients with VSR after MI in our hospital, 88 were included in the analysis (Figure 1). The average age was 70.2 years (41–92 years), and 49 patients (55.7%) were male. Of the patients included, 60 (68.2%) developed CS within 2 weeks after VSR discovery, including 32 (36.4%) patients who already had CS at the time of VSR discovery, and 28 (31.8%) patients who developed delayed CS (within 2 weeks) despite initial hemodynamic stability. The median time from VSR discovery to CS was 2 days in the delayed CS group. The other 28 patients (31.8%) did not develop CS within 2 weeks after VSR discovery. Overall, 19 patients (21.6%) underwent primary PCI, 37 patients (42.0%) underwent MCS, 29 patients (33.0%) underwent ventricular septal repair, 34 patients (38.6%) survived for ≥30 days after discharge, and 54 patients (61.4%) had VSR discovered after admission to our hospital. The median time from MI to VSR discovery was 3 days, and the median time from MI to admission to our hospital was 4 days.

### Characteristics of patients in the immediate CS, delayed CS, and non-CS group

The characteristics of the patients in the immediate CS, delayed CS, and non-CS groups are shown in Table 1. There were statistically significant differences in age, history of smoking, height, SBP, heart rate, left ventricular end-diastolic diameter (LVEDD), creatine kinase-MB isoenzyme, troponin-T, N-terminal pro-B type natriuretic peptide (NT-proBNP), creatinine, survival rates, proportions of inotrope use, MCS, ventricular septal repair, VSR discovered after admission, time from MI to admission to our hospital, time from MI to VSR discovery, and days of hospitalization among the three groups. Compared with patients in the non-immediate CS group, those in the immediate CS group were older and shorter, had lower SBP and DBP levels, higher troponin-T and creatinine levels, smaller LVEDD, lower proportions of smoking, ventricular septal repair and survival, higher proportions of MCS and VSR discovered after admission, shorter time from MI to admission to our hospital, shorter time from MI to VSR discovery, and shorter days of hospitalization. Patients in the delayed CS group had a higher heart rate, higher CK-MB, troponin-T, and NT-proBNP levels, higher proportion of inotrope use, lower survival rates, and shorter time from MI to admission to our hospital than those in the non-CS group.

**Table 1:** The characteristics of the patients in the immediate CS, delayed CS, and non-CS group.

Variables	Total (*n* = 88)	Immediate CS (*n* = 32)	Non-immediate CS (*n* = 56)		*P*1-value^a^	*P*2-value^b^
			Delayed CS (*n* = 28)	Non-CS (*n* = 28)		
Age (years)	70.2 ± 10.8	74.3 ± 7.2*^,^**	70.9 ± 10.6	64.7 ± 12.4	0.006	0.002
Male	49 (55.7%)	14 (43.8%)	15 (53.6%)	20 (71.4%)	0.089	0.095
History of smoking	34 (38.6%)	6 (18.8%)*	12 (42.9%)	16 (57.1%)	0.004	0.008
Height (cm)	161.4 ± 7.8	158.4 ± 7.1*	162.1 ± 7.1	164.1 ± 8.1	0.005	0.012
Weight (kg)	62.4 ± 11.8	59.2 ± 10.2	65.2 ± 14.1	63.0 ± 10.4	0.106	0.228
BMI (kg/cm^2^)	23.9 ± 3.4	23.6 ± 3.4	24.8 ± 4.3	23.4 ± 2.5	0.533	0.333
SBP (mmHg)	105.7 ± 20.0	97.5 ± 19.9*^,^**	110.0 ± 22.5	110.9 ± 14.1	0.003	0.012
DBP (mmHg)	70.5 ± 15.6	65.8 ± 15.9	73.4 ± 16.5	73.0 ± 13.3	0.032	0.102
Heart rate (beats/min)	104.9 ± 18.0	105.4 ± 20.0	111.2 ± 18.1*	98.1 ± 13.2	0.847	0.024
Hypertension	46 (52.3%)	16 (50.0%)	14 (50.0%)	16 (57.1%)	0.747	0.823
Diabetes	28 (31.8%)	11 (34.4%)	6 (21.4%)	11 (39.3%)	0.697	0.331
History of CAD	6 (6.8%)	2 (6.3%)	–	4 (14.3%)	1.000	0.112
Pneumonia	59 (67.0%)	20 (62.5%)	18 (64.3%)	21 (75.0%)	0.493	0.549
Anterior MI	72 (81.8%)	25 (78.1%)	24 (85.7%)	23 (82.1%)	0.497	0.740
LVEDD (mm)	49.6 ± 7.0	46.3 ± 6.0*^,^**	50.5 ± 3.6	52.3 ± 9.1	0.001	0.002
LVEF (%)	48.9 ± 12.2	49.5 ± 11.4	48.9 ± 13.5	48.3 ± 12.1	0.747	0.930
Thickness of IVS (mm)	10.2 ± 2.1	10.5 ± 2.3	10.4 ± 2.4	9.6 ± 1.5	0.507	0.515
Thickness of LVPW (mm)	9.2 ± 1.3	9.3 ± 1.3	9.3 ± 1.4	9.0 ± 1.1	0.499	0.506
Size of VSR (mm)	13.5 ± 5.6	14.3 ± 4.8	13.6 ± 5.9	12.6 ± 6.3	0.314	0.496
VSR location						
Anterior	16 (18.2%)	5 (15.6%)	7 (25.0%)	4 (14.3%)	0.895	0.799
Posterior	24 (27.3%)	9 (27.3%)	6 (21.4%)	9 (32.1%)		
Apical	48 (54.5%)	18 (56.3%)	15 (53.6%)	15 (53.6%)		
Tricuspid regurgitation	16 (18.2%)	7 (21.9%)	6 (21.4%)	3 (10.7%)	0.497	0.455
CK-MB (ng/mL)	65.6 ± 93.03	88.6 ± 96.4*	91.9 ± 109.1*	13.1 ± 35.7	0.080	0.001
Troponin-T (ng/mL)	3.56 ± 3.27	5.01 ± 3.58*	4.00 ± 3.03*	1.45 ± 1.81	0.001	<0.001
NT-proBNP (μg/L)	13.22 ± 11.61	15.94 ± 12.68*	14.70 ± 11.86*	8.62 ± 8.77	0.097	0.035
Creatinine (μmol/L)	138.4 ± 73.2	168.8 ± 83.2*	137.8 ± 74.5	104.3 ± 38.2	0.003	0.002
Total cholesterol (mmol/L)	4.02 ± 1.18	3.95 ± 1.22	4.32 ± 1.21	3.80 ± 1.08	0.672	0.242
HDL-C (mmol/L)	1.06 ± 0.40	1.12 ± 0.36	1.10 ± 0.46	0.93 ± 0.35	0.234	0.129
LDL-C (mmol/L)	2.50 ± 0.96	2.45 ± 0.97	2.74 ± 1.00	2.32 ± 0.88	0.715	0.257
Primary PCI	19 (21.6%)	6 (18.8%)	7 (25.0%)	6 (21.4%)	0.624	0.841
Preoperative PCI	39 (44.3%)	18 (56.3%)	9 (28.6%)	13 (46.4%)	0.089	0.095
Angiographic data						
One-vessel disease	41 (64.1%)	13 (59.1%)	11 (68.8%)	17 (65.4%)	0.845	0.961
Two-vessel disease	17 (26.6%)	7 (31.8%)	4 (25.0%)	6 (23.1%)		
Three-vessel disease	6 (9.4%)	2 (9.1%)	1 (6.3%)	3 (11.5%)		
Killip class						
I	3 (3.4%)	–	–	3 (10.7%)	<0.001	<0.001
II	25 (28.4%)	–	11 (39.3%)	14 (50.0%)		
III	30 (34.1%)	2 (6.3%)	17 (60.7%)	11 (39.3%)		
IV	30 (34.1%)	30 (93.8%)	–	–		
Inotrope use	69 (78.4%)	29 (90.6%)*	25 (89.3%)*	15 (53.6%)	0.057	0.001
MCS	37 (42.0%)	21 (65.6%)*	10 (35.7%)	6 (21.4%)	0.001	0.002
Ventricular septal repair	29 (33.0%)	5 (15.6%)*	9 (32.1%)	15 (53.6%)	0.009	0.007
Survived	34 (38.6%)	5 (15.6%)*	8 (28.6%)*	21 (75.0%)	0.001	<0.001
VSR discovered after admission	54 (61.4%)	28 (87.5%)*	17 (60.7%)	9 (32.1%)	<0.001	<0.001
Time from MI to VSR discovery (days)	3 (1, 6.75)	2 (0.685,4)*	4.25 (1, 6)	5 (1, 11.5)	0.018	0.032
Time from MI to admission to our hospital (days)	4 (1, 7.75)	1.73 (0.685, 4)*	2.5 (0.87, 7)*	9 (5.125, 13.5)	<0.001	<0.001
Days of hospitalization (days)	9 (2, 20)	2 (1, 12)*^,^**	8 (3.25, 23)	17 (10.25, 21.75)	0.002	0.004
Time from VSR to CS (days)	–	–	2 (1, 5.75)	–	–	–

Continuous data are presented as mean ± SD or median (IQR), as appropriate. Categorical data are presented as *n* (%). Echocardiographic data were obtained when VSR was first detected in our hospital, and hematological data were obtained on admission.

CS, cardiogenic shock; BMI, body mass index; SBP, systolic blood pressure; DBP, diastolic blood pressure; CAD, coronary artery disease; MI, acute myocardial infarction; LVEDD, left ventricular end-diastolic diameter; LVEF, left ventricular ejection fraction; IVS, interventricular septum; LVPW, left ventricular posterior wall; VSR, ventricular septal rupture; CK-MB, creatine kinase-MB isoenzyme; NT-proBNP, N-terminal pro-B type natriuretic peptide; HDL-C, high-density lipoprotein cholesterol; LDL-C, low-density lipoprotein cholesterol; PCI, percutaneous coronary intervention; MCS, mechanical circulatory support.

* *P* < 0.05 vs. non-CS group.

** *P* < 0.05 vs. delayed CS group.

^a^ Comparison between the immediate CS and non-immediate CS groups.

^b^ Comparison among the three groups.

### Logistic regression analysis of the risk factors for immediate CS in patients with VSR after MI

Table 2 demonstrates that age, sex, and variables with a statistical correlation with immediate CS in patients with VSR after MI in the univariate logistic regression analysis, which may affect the occurrence of immediate CS in clinical settings, were included in the multivariable logistic regression analysis. These variables included history of smoking, height, LVEDD, troponin-T levels, VSR discovered after admission, and the time from MI to admission to our hospital. Variables affected by CS itself, including SBP, DBP, and creatinine levels, were excluded. MCS and ventricular septal repair that were generally performed after the onset of CS were also excluded. Multivariable regression analysis (forward: LR) indicated that a smaller LVEDD (OR: 0.849, 0.759–0.950) and VSR discovered after admission (OR: 7.401, 2.155–25.423) were independent risk factors for the occurrence of immediate CS in patients with VSR after MI.

**Table 2:** Logistic regression analysis of the risk factors for immediate CS in patients with VSR after MI compared with patients without immediate CS.

Variables	Total subjects (*n* = 88)			
	Univariate analysis		Multivariate analysis	
	OR (95% CI)	*P*-value	OR (95% CI)	*P*-value
Age (years)	1.066 (1.016–1.118)	0.009		
Sex (male)	0.467 (0.193–1.129)	0.091		
History of smoking	0.231 (0.082–0.647)	0.005		
Height (cm)	0.915 (0.856–0.977)	0.008		
LVEDD (mm)	0.839 (0.757–0.930)	0.001	0.849 (0.759–0.950)	0.004
Troponin-T (ng/mL)	1.246 (1.081–1.436)	0.002		
VSR discovered after admission	8.077 (2.502–26.073)	<0.001	7.401 (2.155–25.423)	0.001
Time from MI to admission to our hospital (days)	0.895 (0.811–0.988)	0.027		

OR, odds ratio; CI, confidence interval; CS, cardiogenic shock; VSR, ventricular septal rupture; LVEDD, left ventricular end-diastolic diameter; MI, myocardial infarction.

### Logistic regression analysis of the risk factors for delayed CS in patients without immediate CS after VSR

Table 3 shows that age, sex, and variables with a statistical correlation with delayed CS in patients without immediate CS after VSR in the univariable regression logistic analysis, which may affect the occurrence of delayed CS in clinical settings, were included in the multivariable logistic regression analysis, such as heart rate, levels of CK-MB, troponin-T and NT-proBNP, VSR discovered after admission, and the time from MI to admission to our hospital. Inotropes were sometimes administered following the onset of CS in the delayed CS group; therefore, inotrope use was not included in the logistic analysis. Multivariate regression analysis (forward: LR) indicated that an elevated heart rate (OR: 1.084, 1.025–1.146) and higher CK-MB levels (OR: 1.021, 1.007–1.036) on admission were independent risk factors for the occurrence of delayed CS in patients without immediate CS after VSR.

**Table 3:** Logistic regression analysis of the risk factors for delayed CS in patients without immediate CS after VSR.

Variables	Total subjects (*n* = 56)			
	Univariate analysis		Multivariate analysis	
	OR (95% CI)	*P*-value	OR (95% CI)	*P*-value
Age (years)	1.049 (0.999–1.102)	0.055		
Sex (male)	0.462 (0.153–1.395)	0.171		
Heart rate (beats/min)	1.056 (1.014–1.099)	0.008	1.084 (1.025–1.146)	0.005
CK-MB (ng/mL)	1.017 (1.003–1.031)	0.016	1.021 (1.007–1.036)	0.004
Troponin-T (ng/mL)	1.573 (1.172–2.111)	0.003		
NT-proBNP (μg/L)	1.061 (1.002–1.123)	0.044		
VSR discovered after admission	3.263 (1.089–9.776)	0.035		
Time from MI to admission to our hospital (days)	0.839 (0.742–0.950)	0.006		

OR, odds ratio; CI, confidence interval; CS, cardiogenic shock; VSR, ventricular septal rupture; CK-MB, creatine kinase-MB isoenzyme; NT-proBNP, N-terminal pro-B type natriuretic peptide; MI, myocardial infarction.

## Discussion

Cardiogenic shock is a common complication of VSR after MI and an important risk factor for poor prognosis of VSR (7, 9, 10). The reported incidence rate of CS after VSR ranges from 51.7% to 71% (1, 5, 14, 15), and the incidence of CS observed in our study was similar to that reported in previous studies. Our study also indicated the time course for the occurrence of CS after VSR. A patient may have CS immediately at the time of VSR discovery or develop delayed CS while waiting for VSR repair despite hemodynamic stability at the beginning after VSR discovery. An early identification of patients at risk of immediate or delayed CS and optimization of treatment procedures may help improve the prognosis.

The LVEDD is usually obtained in the parasternal long-axis section. It is a simple index commonly used to evaluate the size of the left ventricle (LV). The size of LVEDD in adults is affected by age, sex, height, weight, and heart disease status (16, 17). LV dilation is generally considered a predictor of poor prognosis in patients with heart failure (18). However, our study indicated that patients who developed CS immediately following VSR had the smallest LVEDDs, and a smaller LVEDD was an independent risk factor for immediate CS. This phenomenon was also observed in a study conducted by Hua et al. (19) that patients who died of VSR after MI had smaller LVEDDs than those who survived. Although a longer time from MI to admission to our hospital in the non-immediate CS group might cause LV dilation (20), it may also be related to specific hemodynamic changes in patients with VSR after MI. VSR leads to a new left-to-right shunt, increases pulmonary blood flow, and secondarily increases blood volume back to the LV (21). In patients with congenital ventricular septal defect, the LV gradually expands to adapt to the increased left ventricular blood volume in the process of growth and development. However, in the case of sudden VSR, the LV may not expand sufficiently to accommodate the increased blood volume back to the LV in a short time, combined with reduced left ventricular systolic function, leading to pulmonary congestion, reduced cardiac output, and even CS. However, in a patient with atrial fibrillation who developed CS immediately after the implantation of a left atrial appendage occluder in our center (22), we observed an adaptive expansion of the left atrium in a short time. We believe that whether the LV can dilate adaptively to increase cardiac output and reduce pulmonary edema in the short term after VSR is a very important factor in predicting whether patients will develop CS immediately following the occurrence of VSR. Owing to the lack of LVEDD data prior to the VSR, the effect of LVEDD changes before and after the VSR on the occurrence of CS could not be clarified; thus, this hypothesis needs further verification. In addition, among patients with immediate CS, 87.5% exhibited a VSR after admission, and a VSR discovered after admission was also an independent risk factor for immediate CS. This may be related to a selection bias. Our hospital was not always the first hospital that patients attended at the time of an acute MI attack, and most patients were transferred from other hospitals due to treatment difficulties or mechanical complications such as VSR after acute MI. Patients with immediate CS after VSR in another hospital may not have been transferred to our hospital for further treatment because of their poor condition. Therefore, most of the VSRs in patients with immediate CS were discovered in our hospital.

For patients who develop CS immediately after VSR discovery, the treatment should be aggressive, including vasoactive medications, IABP, and other MCS when necessary, to prolong the waiting time prior to repair (7, 10–12). If the hemodynamics remain unstable with MCS, emergency repair should be conducted although there is a high mortality rate (7, 23). However, in our study population with immediate CS, the proportions of MCS and repair were all lower than those in foreign studies (2, 5, 24), and the rate of surviving ≥30 days after discharge was only 15.6%. The patient's poor condition on admission may lead to patient's family members being pessimistic about the treatment outcome and giving up active treatment owing to limited economic conditions. The optimal treatment procedure for patients without immediate CS after VSR remains unclear. Hobbs et al. (25) have suggested that the IABP should be implanted immediately after VSR in all patients. However, we consider that for patients who do not have immediate CS but have a high risk of developing delayed CS, prophylactic implantation of an IABP may help reduce the occurrence of CS and improve the prognosis. On the other hand, for patients who have a low risk of developing CS after VSR, the benefits of IABP may be limited due to the possibility of complications related to IABP implantation. Therefore, it is particularly important to identify the risk factors for delayed CS in patients who do not have CS immediately at the time of VSR discovery.

Elevated heart rate is an important risk factor for poor prognosis after MI (26). Both animal and clinical studies have shown that elevated heart rate is associated with increased infarct size (27, 28). Elevated heart rate was also associated with a higher risk of malignant arrhythmias in patients with acute MI and reduced ejection fraction (29). In patients with VSR after MI, tachycardia is an independent risk factor for in-hospital mortality (19). In the case of decreased cardiac function, elevated heart rate itself is a compensatory response to maintain a stable cardiac output and sufficient perfusion of the heart and other vital organs (30). In our study, elevated heart rate on admission was an independent risk factor for developing delayed CS in patients who did not have CS immediately at the time of VSR discovery. This suggests that elevated heart rate is not only a result of decreased cardiac function, but also an important cause of the occurrence of CS in patients with VSR after AMI. This is consistent with the discovery by Nepper-Christensen et al. (28) that elevated heart rate in patients with ST-segment elevation MI may be a cause, not a consequence, of larger myocardial damage. In patients with VSR and stable initial hemodynamics, elevated heart rate indicates insufficient cardiac stroke output or other concomitant factors that lead to tachycardia, such as infection, anemia, or excessively increased sympathetic excitability. A compensatory increase in heart rate may maintain circulatory stability for a period of time, but the elevated heart rate will lead to an increase in myocardial oxygen consumption, and a decrease in oxygen supply due to a shortened diastolic period (31). If the cause cannot be corrected in time or no further hemodynamic support is provided, it may develop into decompensation, leading to the occurrence of CS. This finding provides an easy tool to identify patients who do not have CS immediately at the time of VSR discovery, but have a high risk of developing delayed CS, which is particularly important for guiding clinical practice. Further research is needed on how much an increase in the heart rate may have harmful effects and on interventions.

CK-MB level is a specific and sensitive indicator of myocardial injury (32). It can rise 3–4 h after the onset of MI symptoms, reach its peak after 12–24 h, and usually return to baseline values after 48–72 h (32). Peak CK-MB level has been shown to correlate well with infarct size (33, 34). Therefore, the level of CK-MB on admission correlated with the time from MI to admission to our hospital and infarct size. However, the median time from MI to admission to our hospital was 2.5 days in the delayed CS group and 9 days in the non-CS group, both exceeding the peak time of CK-MB. Moreover, echocardiography showed no significant difference in left ventricular ejection fraction (LVEF) between the delayed CS and non-CS groups, suggesting that the effect of higher CK-MB level on the occurrence of delayed CS may be mainly related to the difference in the time from MI to admission to our hospital, rather than the difference in infarct size. Univariate regression analysis showed the effect of time from MI to admission to our hospital on delayed CS. The shorter the time from MI to admission to our hospital, the greater the risk of delayed CS. This is inconsistent with common view that the time from symptom onset to first medical contact is significantly associated with MI prognosis, that is, the shorter the time from MI onset to the first medical contact, the better the prognosis (35, 36). However, since delayed reperfusion itself is an important risk factor for VSR after MI (7), and most patients with VSR after MI have the problem of delayed medical contact, this concept does not seem to be applicable in patients with VSR. In addition, the time from MI to admission in our study referred to the time from the onset of acute chest pain to admission to our hospital rather than the time from symptom onset to first medical contact. Some decompensated patients might die in another hospital or were not eligible to be transferred to our hospital, resulting in a selection bias. Therefore, caution should be exercised when generalizing this conclusion. However, this conclusion remains significant for clinical practice.

Our study had several limitations. First, this was a retrospective study from a single center, which is not always the first hospital to treat patients with acute chest pain, leading to selection bias. This may not fully reflect the overall occurrence of CS in all patients with VSR after MI, and the promotion of research results needs to be performed with caution. However, this study may reflect the basic characteristics of such patients in tertiary hospitals and provide references for management strategies. Second, owing to the limitations of retrospective studies, we were unable to determine the exact time at which CS occurred. Instead, the occurrence of CS was determined by two experienced physicians based on the BP, clinical manifestations, lactate levels, and medication use in medical records. The CS onset time was defined as the time at which catecholamines were initiated. Although there may be deviations, it can reflect the onset time of CS as judged by physicians at that time. Third, we may not have been able to find VSR at the first time when it occurred, and the chronological sequence of CS and VSR could not be definitively defined in patients in the immediate CS group. To avoid bias, we excluded patients who did not have VSR at the time of CS occurred but develop VSR subsequently, and those patients who were found to have VSR at the time of CS occurred were included in the immediate CS group.

## Conclusions

Our study indicates that the occurrence of CS in patients with VSR after MI has an evident time course. Smaller LVEDD and VSR discovered after admission are independent risk factors for immediate CS. In addition, elevated heart rate and higher CK-MB levels on admission are independent risk factors for delayed CS in patients without immediate CS after VSR. An early identification of patients at risk of immediate CS or delayed CS and optimization of treatment procedures may help improve the prognosis. Prospective cohort studies with larger sample sizes are required to verify our conclusions.

## Data Availability

The raw data supporting the conclusions of this article will be made available by the authors, without undue reservation.

## References

[1] Crenshaw BS Granger CB Birnbaum Y Pieper KS Morris DC Kleiman NS Risk factors, angiographic patterns, and outcomes in patients with ventricular septal defect complicating acute myocardial infarction. GUSTO-I (Global Utilization of Streptokinase and TPA for Occluded Coronary Arteries) Trial Investigators. Circulation. (2000) 101:27–32. 10.1161/01.cir.101.1.27 10618300

[2] Moreyra AE Huang MS Wilson AC Deng Y Cosgrove NM Kostis JB Trends in incidence and mortality rates of ventricular septal rupture during acute myocardial infarction. Am J Cardiol. (2010) 106:1095–100. 10.1016/j.amjcard.2010.06.013 20920645

[3] Menon V Webb JG Hillis LD Sleeper LA Abboud R Dzavik V Outcome and profile of ventricular septal rupture with cardiogenic shock after myocardial infarction: a report from the SHOCK Trial Registry. SHould we emergently revascularize Occluded Coronaries in cardiogenic shocK? J Am Coll Cardiol. (2000) 36:1110–6. 10.1016/s0735-1097(00)00878-0 10985713

[4] Poulsen SH Praestholm M Munk K Wierup P Egeblad H Nielsen-Kudsk JE. Ventricular septal rupture complicating acute myocardial infarction: clinical characteristics and contemporary outcome. Ann Thorac Surg. (2008) 85:1591–6. 10.1016/j.athoracsur.2008.01.010 18442545

[5] Arnaoutakis GJ Zhao Y George TJ Sciortino CM McCarthy PM Conte JV. Surgical repair of ventricular septal defect after myocardial infarction: outcomes from the Society of Thoracic Surgeons National Database. Ann Thorac Surg. (2012) 94:436–43. 10.5090/kjtcs.2013.46.6.433 22626761 PMC3608099

[6] Chinese Society of Cardiology of Chinese Medical Association, Editorial Board of Chinese Journal of Cardiology. 2019 Chinese Society of Cardiology (CSC) guidelines for the diagnosis and management of patients with ST-segment elevation myocardial infarction. Zhonghua Xin Xue Guan Bing Za Zhi. (2019) 47:766–83. 10.3760/cma.j.issn.0253-3758.2019.10.003 31648459

[7] Damluji AA van Diepen S Katz JN Menon V Tamis-Holland JE Bakitas M Mechanical complications of acute myocardial infarction: a scientific statement from the American Heart Association. Circulation. (2021) 144:e16–35. 10.1161/CIR.0000000000000985 34126755 PMC9364424

[8] Rashid H Kumar K Ullah A Kamin M Shafique HM Elahi A Delayed ventricular septal rupture repair on patient outcomes after myocardial infarction: a systematic review. Curr Probl Cardiol. (2023) 48:101521. 10.1016/j.cpcardiol.2022.101521 36455796

[9] Elbadawi A Elgendy IY Mahmoud K Barakat AF Mentias A Mohamed AH Temporal trends and outcomes of mechanical complications in patients with acute myocardial infarction. JACC Cardiovasc Interv. (2019) 12:1825–36. 10.1016/j.jcin.2019.04.039 31537282

[10] Zbikowska K Wrobel K. Mechanical circulatory support in delayed surgery of post-infarction ventricular septal rupture in patients in cardiogenic shock—a review. J Clin Med. (2022) 11:4728. 10.3390/jcm11164728 36012967 PMC9409930

[11] Zeymer U Bueno H Granger CB Hochman J Huber K Lettino M Acute Cardiovascular Care Association position statement for the diagnosis and treatment of patients with acute myocardial infarction complicated by cardiogenic shock: a document of the Acute Cardiovascular Care Association of the European Society of Cardiology. Eur Heart J Acute Cardiovasc Care. (2020) 9:183–97. 10.1177/2048872619894254 32114774

[12] Chioncel O Parissis J Mebazaa A Thiele H Desch S Bauersachs J Epidemiology, pathophysiology and contemporary management of cardiogenic shock—a position statement from the Heart Failure Association of the European Society of Cardiology. Eur J Heart Fail. (2020) 22:1315–41. 10.1002/ejhf.1922 32469155

[13] Omar S Morgan GL Panchal HB Thourani V Rihal CS Patel R Management of post-myocardial infarction ventricular septal defects: a critical assessment. J Interv Cardiol. (2018) 31:939–48. 10.1111/joic.12556 30318677

[14] Cinq-Mars A Voisine P Dagenais F Charbonneau E Jacques F Kalavrouziotis D Risk factors of mortality after surgical correction of ventricular septal defect following myocardial infarction: retrospective analysis and review of the literature. Int J Cardiol. (2016) 206:27–36. 10.1016/j.ijcard.2015.12.011 26773765

[15] Wang L Xiao LL Liu C Zhang YZ Zhao XY Li L Clinical characteristics and contemporary prognosis of ventricular septal rupture complicating acute myocardial infarction: a single-center experience. Front Cardiovasc Med. (2021) 8:679148. 10.3389/fcvm.2021.679148 34589525 PMC8473686

[16] Seko Y Kato T Morita Y Yamaji Y Haruna Y Izumi T Age- and body size-adjusted left ventricular end-diastolic dimension in a Japanese hospital-based population. Circ J. (2019) 83:604–13. 10.1253/circj.CJ-18-1095 30700662

[17] Echocardiographic Normal Ranges Meta-Analysis of the Left Heart Collaboration. Ethnic-specific normative reference values for echocardiographic LA and LV size, LV mass, and systolic function: the EchoNoRMAL study. JACC Cardiovasc Imaging. (2015) 8:656–65. 10.1016/j.jcmg.2015.02.014 25981507

[18] McDonagh TA Metra M Adamo M Gardner RS Baumbach A Bohm M 2021 ESC guidelines for the diagnosis and treatment of acute and chronic heart failure. Eur Heart J. (2021) 42:3599–726. 10.1093/eurheartj/ehab368 34447992

[19] Hua K Peng Z Yang X. Long-term survival and risk factors for post-infarction ventricular septal rupture. Heart Lung Circ. (2021) 30:978–85. 10.1016/j.hlc.2020.11.013 33495129

[20] Korup E Dalsgaard D Nyvad O Jensen TM Toft E Berning J. Comparison of degrees of left ventricular dilation within three hours and up to six days after onset of first acute myocardial infarction. Am J Cardiol. (1997) 80:449–53. 10.1016/s0002-9149(97)00393-7 9285656

[21] Birnbaum Y Fishbein MC Blanche C Siegel RJ. Ventricular septal rupture after acute myocardial infarction. N Engl J Med. (2002) 347:1426–32. 10.1056/NEJMra020228 12409546

[22] Xiao Q Pu X Wei X Chen M. Refractory cardiogenic shock in a senile atrial fibrillation patient after left atrial appendage occlusion. Eur Heart J. (2022) 43:4663. 10.1093/eurheartj/ehac565 36245073

[23] Ronco D Matteucci M Kowalewski M De Bonis M Formica F Jiritano F Surgical treatment of postinfarction ventricular septal rupture. JAMA Netw Open. (2021) 4:e2128309. 10.1001/jamanetworkopen.2021.28309 34668946 PMC8529403

[24] Sakaguchi G Miyata H Motomura N Ueki C Fukuchi E Yamamoto H Surgical repair of post-infarction ventricular septal defect—findings from a Japanese national database. Circ J. (2019) 83:2229–35. 10.1253/circj.CJ-19-0593 31511450

[25] Ronco D Matteucci M Ravaux JM Marra S Torchio F Corazzari C Mechanical circulatory support as a bridge to definitive treatment in post-infarction ventricular septal rupture. JACC Cardiovasc Interv. (2021) 14:1053–66. 10.1016/j.jcin.2021.02.046 34016403

[26] Antoni ML Boden H Delgado V Boersma E Fox K Schalij MJ Relationship between discharge heart rate and mortality in patients after acute myocardial infarction treated with primary percutaneous coronary intervention. Eur Heart J. (2012) 33:96–102. 10.1093/eurheartj/ehr293 21862462

[27] Maroko PR Kjekshus JK Sobel BE Watanabe T Covell JW Ross J Factors influencing infarct size following experimental coronary artery occlusions. Circulation. (1971) 43:67–82. 10.1161/01.cir.43.1.67 5540853

[28] Nepper-Christensen L Lonborg J Ahtarovski KA Hofsten DE Kyhl K Schoos MM Importance of elevated heart rate in the very early phase of ST-segment elevation myocardial infarction: results from the DANAMI-3 trial. Eur Heart J Acute Cardiovasc Care. (2019) 8:318–28. 10.1177/2048872618795515 30136597

[29] Lai M Cheung CC Olgin J Pletcher M Vittinghoff E Lin F Risk factors for arrhythmic death, overall mortality, and ventricular tachyarrhythmias requiring shock after myocardial infarction. Am J Cardiol. (2023) 187:18–25. 10.1016/j.amjcard.2022.10.009 36459743

[30] Reynolds HR Hochman JS. Cardiogenic shock: current concepts and improving outcomes. Circulation. (2008) 117:686–97. 10.1161/CIRCULATIONAHA.106.613596 18250279

[31] Fox K Borer JS Camm AJ Danchin N Ferrari R Lopez Sendon JL Resting heart rate in cardiovascular disease. J Am Coll Cardiol. (2007) 50:823–30. 10.1016/j.jacc.2007.04.079 17719466

[32] Navin TR Hager WD. Creatine kinase MB isoenzyme in the evaluation of myocardial infarction. Curr Probl Cardiol. (1979) 3:1–32. 10.1016/0146-2806(79)90010-0 389571

[33] Grande P Hansen BF Christiansen C Naestoft J. Estimation of acute myocardial infarct size in man by serum CK-MB measurements. Circulation. (1982) 65:756–64. 10.1161/01.cir.65.4.756 7060254

[34] Poyhonen P Kylmala M Vesterinen P Kivisto S Holmstrom M Lauerma K Peak CK-MB has a strong association with chronic scar size and wall motion abnormalities after revascularized non-transmural myocardial infarction—a prospective CMR study. BMC Cardiovasc Disord. (2018) 18:27. 10.1186/s12872-018-0767-7 29422025 PMC5806273

[35] Ibanez B James S Agewall S Antunes MJ Bucciarelli-Ducci C Bueno H 2017 ESC guidelines for the management of acute myocardial infarction in patients presenting with ST-segment elevation: the Task Force for the management of acute myocardial infarction in patients presenting with ST-segment elevation of the European Society of Cardiology (ESC). Eur Heart J. (2018) 39:119–77. 10.1093/eurheartj/ehx393 28886621

[36] Wei TF Zhao B Liu PL Feng XY Zhang Z Shi QX Impact of symptom onset to first medical contact time on the prognosis of patients with acute ST-segment elevation myocardial infarction. Zhonghua Xin Xue Guan Bing Za Zhi. (2017) 45:393–98. 10.3760/cma.j.issn.0253-3758.2017.05.006 28511323

